# Computer-Aided System Application Value for Assessing Hip Development

**DOI:** 10.3389/fphys.2020.587161

**Published:** 2020-12-01

**Authors:** Yaoxian Jiang, Guangyao Yang, Yuan Liang, Qin Shi, Boqi Cui, Xiaodan Chang, Zhaowen Qiu, Xudong Zhao

**Affiliations:** ^1^Department of Radiology, Affiliated Zhongshan Hospital of Dalian University, Dalian, China; ^2^College of Information and Computer Engineering, Northeast Forestry University, Harbin, China; ^3^Department of Radiology, Union Hospital, Tongji Medical College, Huazhong University of Science and Technology, Wuhan, China; ^4^Department of Clinical Medicine, Zhongshan Clinical College of Dalian University, Dalian, China; ^5^Heilongjiang Tuomeng Technology Co., Ltd., Harbin, China

**Keywords:** hip dysplasia, acetabular dysplasia, computer-aided detection, computer-aided diagnosis, x-ray

## Abstract

**Purpose:**

A computer-aided system was used to semiautomatically measure Tönnis angle, Sharp angle, and center-edge (CE) angle using contours of the hip bones to establish an auxiliary measurement model for developmental screening or diagnosis of hip joint disorders.

**Methods:**

We retrospectively analyzed bilateral hip x-rays for 124 patients (41 men and 83 women aged 20–70 years) who presented at the Affiliated Zhongshan Hospital of Dalian University in 2017 and 2018. All images were imported into a computer-aided detection system. After manually outlining hip bone contours, Tönnis angle, Sharp angle, and CE angle marker lines were automatically extracted, and the angles were measured and recorded. An imaging physician also manually measured all angles and recorded hip development, and Pearson correlation coefficients were used to compare computer-aided system measurements with imaging physician measurements. Accuracy for different angles was calculated, and the area under the receiver operating characteristic (AUROC) curve was used to represent the diagnostic efficiency of the computer-aided system.

**Results:**

For Tönnis angle, Sharp angle, and CE angle, correlation coefficients were 0.902, 0.887, and 0.902, respectively; the accuracies of the computer-aided detection system were 89.1, 93.1, and 82.3%; and the AUROC curve values were 0.940, 0.956, and 0.948.

**Conclusion:**

The measurements of Tönnis angle, Sharp angle, and CE angle using the semiautomatic system were highly correlated with the measurements of the imaging physician and can be used to assess hip joint development with high accuracy and diagnostic efficiency.

## Introduction

As one of the largest active joints in the human body, the hip and its joint structure allow activities of daily living. One of the main causes of osteoarthritis of the hip joint is dysplasia of the hip. If the acetabular surface is too shallow or abnormally inclined, it cannot cover the femoral head well, which results in uneven stress on the hip joint, which over a long period develops into irreversible osteoarthritis. Early diagnosis and appropriate clinical management would save most patients with hip dysplasia from the pain of surgery and also save medical resources. Studies have shown that two important causes of early hip degeneration are dysplasia and acetabular impingement (Leunig and Ganz, 2014). Among them, acetabular dysplasia is believed to be a major cause of osteoarthritis in young adults, because it can have different defects in shape, orientation, and size (Gala et al., 2016).

Moreover, there is a special group of patients with hip dysplasia who have stable hips in infancy, with no obvious abnormalities, but develop hip pain in adulthood. The cause of this phenomenon is not clear, but for these patients, imaging examinations have become indispensable in finding the disease. With the development of imaging technology, imaging methods for hip joint screening, such as computed tomography (CT) and magnetic resonance imaging (MRI), have made great progress (Welton et al., 2018a). Although these advanced imaging methods can dynamically observe the three-dimensional structure of the hip joint, which is more advantageous than x-ray photography, the high cost of MRI and the relatively high radiation dose of CT limit their clinical application. Therefore, x-rays remain a component of the standard of care in the initial evaluation of patients undergoing hip examination (Welton et al., 2018a). However, the daily work of imaging medicine is a huge challenge for medical personnel in both quantity and difficulty. For screening hip joint development, if other auxiliary methods can be used to reduce the working pressure of doctors, without reducing assessment accuracy, then this would most benefit patients.

The term “artificial intelligence” has been in use since the 1960s and was used to observe the specific logo structure of an image (Pesapane et al., 2018). It is a science that uses computers to simulate human thinking and behavior (Hosny et al., 2018; Bi et al., 2019). In recent years, with the rapid development of computer technology, artificial intelligence technology has been applied in medical imaging (Siegel, 2012). The combination of artificial intelligence and medical imaging is considered to be a promising field of development (Kahn, 2017). Among them, computer-aided systems were first proposed in the 1960s for x-ray of chest or breast disease (Lee et al., 2017). Tannast et al. (2008) conducted an initial study on computer-aided measurement software, but their main interest was focused on the three-dimensional correction of pelvic tilt. Nepple et al. (2014) have used computer measurements to study the reliability of parameters related to acetabular impingement, but a major limitation of their studies was the lack of direct comparison with manual measurements. The purpose of this study was to investigate whether computer-aided detection could measure the Tönnis angle, Sharp angle, and center-edge (CE) angle of the hip joint by analyzing the correlation between imaging physician and computer-aided detection measurements. Using the measurement results, the status of hip joint development was assessed; receiver operating characteristic (ROC) curve analysis was used to obtain the diagnostic value of computer-aided detection for hip joint development.

## Materials and Methods

### Objectives of Study

For this study, we retrospectively collected digital bilateral hip x-rays from 124 patients (total 248 hips) from the affiliated Zhongshan Hospital of Dalian University from January 2017 to 2018. Patients included 41 males and 83 females. Patient ages ranged from 20 to 70 years, with an average age of 47.23 ± 12.89 years. This study was approved by the hospital’s ethics committee. Because this study was retrospective, visa-free informed consent of patients was obtained. All patient information was de-identified before data analysis.

Inclusion criteria were (1) adults with closed epiphyses, (2) no history of trauma or no obvious bone abnormalities caused by trauma, (3) no obvious deformity of the hip joint, and (4) standard x-ray photography of double hip joints, with clear images.

Exclusion criteria were (1) adolescents without epiphysis closure, (2) obvious bone deformity or bone defect, (3) forced position caused by various reasons, (4) non-standard x-ray photography of the double hip joints or the image quality was not good.

### Equipment and Parameters

The images were taken at the medical imaging center of our hospital, and routine hip scans were performed by Dr. Ysio (Siemens Healthcare, Germany) and DR R-20 (Shimadzu Corporation). The original x-ray images were stored in a picture archiving and communication system (PACS) in DICOM format. The distance from the x-ray tube to the detector was 115 cm, and the projection direction of the x-ray tube was perpendicular to the inspection bed. X-rays were taken with patients in supine position with their lower limbs extended and rotated 15° inward. The center line was a vertical line from the midpoint of a line connecting the anterior superior iliac spine and the midpoint of a line connecting the superior margin of the symphysis pubis. The upper boundary of the irradiation field was the anterior superior iliac spine, and the lower boundary was the lesser trochanter. Conventional scanning parameters were used (tube voltage 70–90 kV, tube current 150–200 mA).

### Research Method

#### Digital X-Ray Image Processing

**FIGURE 1:**
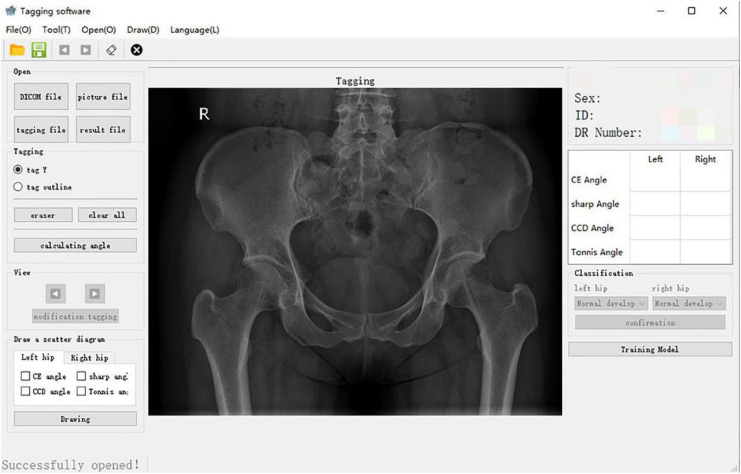
Graphical user interface of the computer-aided detection system.

For patients meeting the inclusion criteria of this study, the original hip joint images were collected, and image information was recorded in an Excel (Microsoft Inc., United States) sheet. This information included gender, age, examination number, and side. The original image was imported into the computer-aided detection system, which mainly included the graphical user interface (Figure 1) for the automatic extraction of angles.

#### Principles of Computer-Aided Measurement

##### Hip joint outlines

**FIGURE 2:**
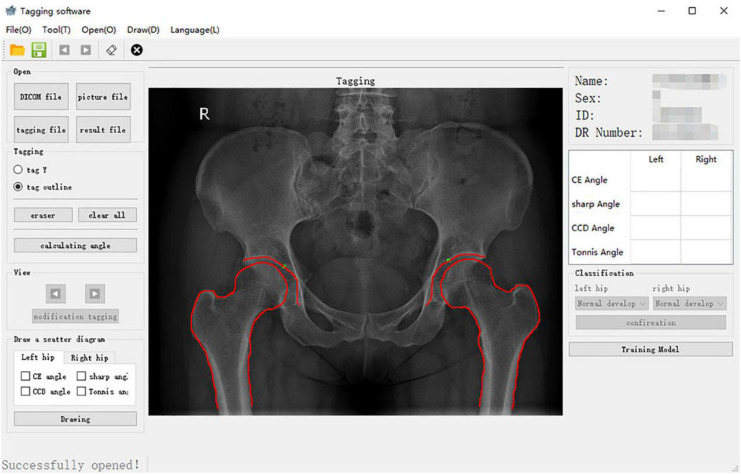
Bony outline of the hip joint. The continuous arcs from the outer upper margin of the acetabulum to the bottom of the acetabular fossa and the femoral head to the neck were traced.

The core of the computer-aided detection system was the semiautomatic extraction of angles. First, an image of the hip joint and bony landmarks (including the femoral head, acetabulum, and pelvic tear drops) was viewed with the graphical user interface. The position of the inside edge of the acetabular weight-bearing area was manually marked. Second, the contours of the hip joint bones were manually traced (Figure 2): (1) the complete arc from the outer edge of the acetabulum to the bottom of the acetabular fossa and (2) from the femoral head to the femoral neck.

##### Automatic extraction of bone landmarks

**FIGURE 3:**
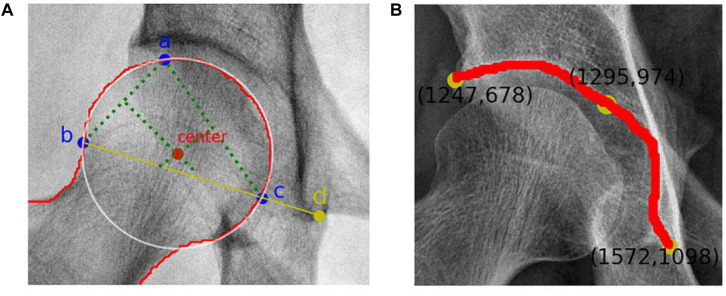
**(A)** Femoral head center recognition. Points a, b, and c are located on the femoral head and are not collinear. The best circle covering the femoral head can be obtained with these three points. The red dot represents the center of the femoral head, and point d is the lower edge of the teardrop. **(B)** Skeletal contour coordinates of the hip joint. The yellow dot on the upper left represents the coordinate value of the outer upper margin of the acetabulum, and the yellow dot on the lower right represents the coordinate value of the lower margin of the teardrop.

The remaining steps were performed automatically by the system. To locate the center of the femoral head, a circle that overlapped with the femoral head to the greatest extent was found. Therefore, geometrically, three points that covered as much of the femoral head as possible and that were not collinear were identified. After repeated tests, these three points were located at (1) the upper part of the femoral head, (2) the lateral part of the femoral head, and (3) the line between the lateral part of the femoral head and the lower edge of the acetabular teardrop on the same side, the intersection of the medial part of the femoral head. The center of the optimal circle was used as the position of the center of the femoral head (Figure 3).

Recognition of the outer upper margin of the acetabulum and the lower margin of the teardrop required processing to obtain a fine contour representation. The coordinates of the bone contours were extracted (Figure 3), and the angular endpoints and vertices were automatically obtained.

##### Automatic angle extraction

**FIGURE 4:**
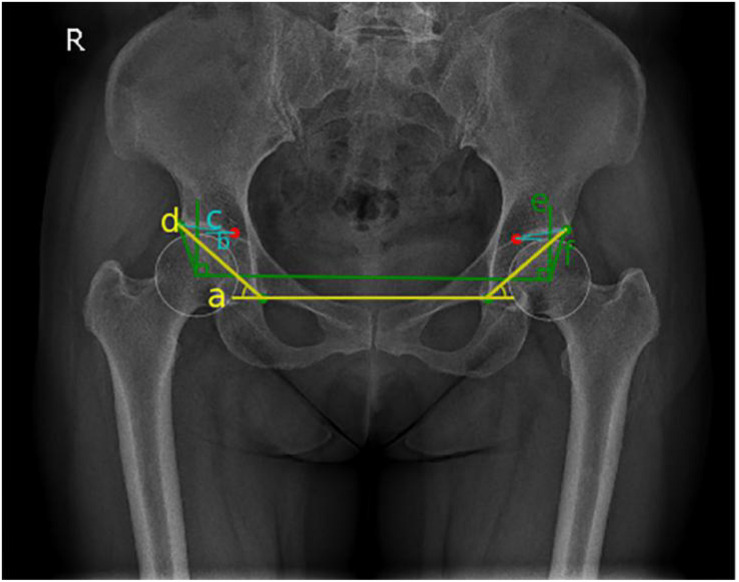
Schematic diagram of hip joint angle measurement: blue represents Tönnis angle, yellow represents Sharp angle, and green represents the center-edge (CE) angle.

**FIGURE 5:**
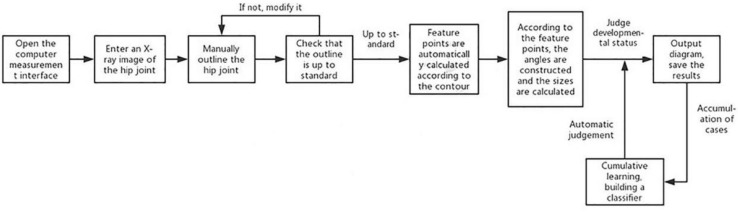
Flowchart of angle measurement by the computer-aided detection system.

Measurement values of each angle were obtained by calculating the vertex angle between vectors. An angle measurement schematic diagram (Figure 4) was displayed on the hip joint image, and the data results were automatically recorded. A flowchart of the relative angles of the hip joint measured using the computer-aided system is shown in Figure 5.

#### Manual Measurement and Reference Values of Each Parameter

Manual measurements were performed by an imaging physician who was certified as a medical practitioner and trained in the musculoskeletal system. In addition, a chief physician with more than 20 years of working experience provided guidance and took the final manual measurements. To confirm the reliability of the measurement results of the imaging physician, reliability analyses of Tönnis angle, Sharp angle, and CE angle were performed. After initial measurement, 80 hip joint images were randomly selected for remeasurement after a 1-month interval, and Cronbach α was calculated. Cronbach α values for Tönnis angle, Sharp angle, and CE angle were 0.992, 0.969, and 0.988, respectively. Measurement consistency of the angle index of hip joint was very high, and the reliability was strong. Based on the manual measurements, the development status of the hip joints was independently assessed.

##### Tönnis angle

On anterior and posterior pelvic x-rays, the lower edges of the teardrops on both sides were connected as the horizontal axis of the pelvis (line a), line b was the inner edge of the weight-bearing area of the acetabulum parallel to line a, and line c connected the inner and outer edges of the weight-bearing region of the acetabulum. The interior angle between line c and line b was the Tönnis angle (Tönnis and Heinecke, 1999; Figure 4). Hip dysplasia is defined as a Tönnis angle greater than 10°.

##### Sharp angle

On anterior and posterior pelvic x-rays, line d through the lower edge of the teardrop is connected to the lateral edge of the acetabulum. The interior angle between line d and line a is the Sharp angle (Figure 4). Hip dysplasia is defined as a Sharp angle greater than 45° (Tannast et al., 2007; Welton et al., 2018b).

##### CE angle

On anterior and posterior pelvic x-rays, vertical line e is formed through the center of the femoral head, and horizontal line f is formed through the center of the femoral head by connecting the outer edges of the acetabulum. The acute angle formed by line e and the line f is called the CE angle (Beltran et al., 2013; Figure 4). Finding the center of femoral head is the key to measuring the CE angle. In manual measurements of CE angle, the PACS was used to make a circle with the maximum overlap with the edge of the femoral head, and the intersection point of the diameter is the center of the circle. Hip dysplasia is defined as a CE angle less than 20°, and CE angles between 20 and 25° were defined as borderline dysplasia. [Femoral head coverage was less than the lower limit, but they were not considered as dysplasia (Wiberg and Frey, 1940)].

#### Preliminary Experiment

**FIGURE 6:**
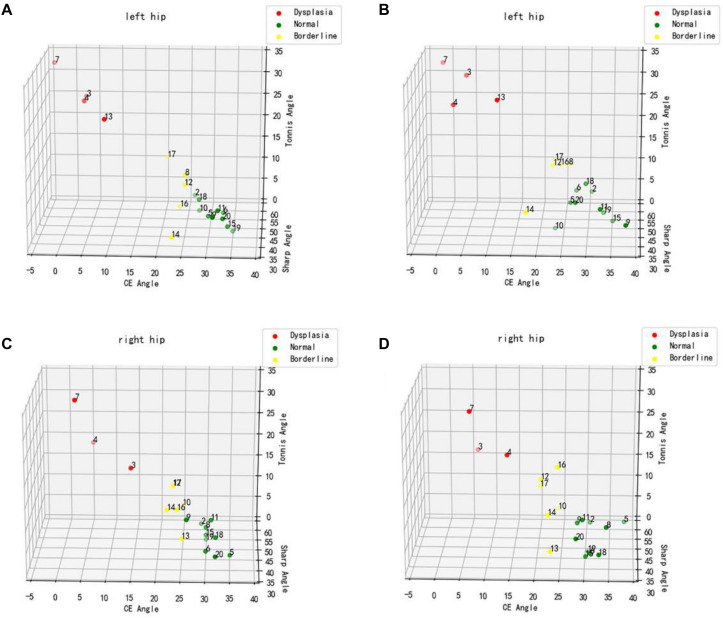
Distribution diagram of hip joint measurement results. **(A,C)** Physician measurement results. **(B,D)** Computer-aided system measurement results.

To verify the feasibility of this study, a preliminary experimental study was conducted on 19 patients who underwent bilateral hip x-rays (total of 38 hips) in Zhongshan Hospital of Dalian University. The Tönnis angle, Sharp angle, and CE angle of all hip joints were measured by an imaging physician and the computer-aided system. The CE angle was selected to assess and classify the development status of all hip joints, and the 19 patients were labeled with colors (red represented dysplasia, green represented normal development, and yellow represented patients with marginal dysplasia). Three-dimensional scatter plots were drawn to show the distribution diagram of the hip joint measurement results by imaging physician and computer-aided system (Figure 6). From the distribution diagram of the preliminary experiment results, the computer-aided system could distinguish patients with dysplasia and those with normal development, but there were also some deficiencies. For a few patients at the boundary between normal development and dysplasia, the computer assessments were incorrect.

#### Statistical Analysis

SPSS (version 25.0; IBM Corp.) software was used for statistical analysis and processing. Enumeration data were expressed by mean value and standard deviation, and descriptive analysis and correlation analysis were carried out. The measurements by imaging physicians were used as the reference standard, independent assessments were made with each angle, and the development status of each hip joint was recorded. A machine learning classification model – support vector machine (SVM) – assessed and classified hip joint development using the measurement results from the automatic calculation. Our whole system was developed using Python 3. Module Scikit-learn was used to present SVM for classification. Here, nonlinear SVM was adopted. Meanwhile, a polynomial kernel was utilized with its degree of determined to be 2. Its penalty coefficient was set to 0.3. The rest parameters were set to default values. The accuracy, specificity, and sensitivity of computer-aided detection in assessing the development of the hip joint under different reference standards were calculated based on the semiautomatic measurements. Accuracy was the ratio of the number of cases of hip joint development correctly assessed by computer-aided detection to the total number of cases of hip joint. Specificity (true negative rate) was the ratio of the number of cases with normal hip joint development correctly assessed by computer-aided detection to the total number of cases with normal hip joint development. Sensitivity (true positive rate) was the ratio of the number of cases of hip dysplasia correctly determined by computer-aided detection to the total number of cases of hip dysplasia. The McNemar test was used to compare the differences between the computer-aided system and the imaging physician in assessing hip joint development. The ROC curve was used to analyze measurements of various angles for the diagnostic value of computer-aided detection in assessing hip joint development. The area under the receiver operating characteristic (AUROC) curve was calculated to quantify its diagnostic efficiency. The test level was set as *P* = 0.05, and *P* < 0.05 was considered statistically significant.

## Results

In this study, 248 hip joint digital x-ray images were included. The measurement indices of hip joint included Tönnis angle, Sharp angle, and CE angle. For each angle, the development status of hip joint was independently assessed and recorded.

### Imaging Physician and Computer Measurements

Descriptive statistical results of the imaging physician and the computer measurements are shown in Table 1. The average values of Tönnis angle, Sharp angle, and CE angle measured by the imaging physician were 8.490° (range, −8.000 to 33.000°), 39.897° (range, 30.600 to 55.600°), and 28.129° (range, −4.000 to 47.000°), respectively. Average values of Tönnis angle, Sharp angle, and CE angle measured by computer were 7.967° (range, −7.770 to 33.050°), 40.885° (range, 27.840 to 56.182°), and 26.837° (range, −1.625 to 44.350°), respectively.

**TABLE 1:** Details of the hip angle measurements from the x-ray images.

Angle	Method	Maximum	Minimum	Mean	Standard deviation	SEM
Tönnis	Physician measurements	33.000	−8.000	8.490	6.510	0.413
	CAD measurements	33.050	−7.770	7.967	7.268	0.462
Sharp	Physician measurements	55.600	30.600	39.897	4.178	0.265
	CAD measurements	56.182	27.840	40.885	4.576	0.291
CE	Physician measurements	47.000	−4.000	28.129	8.090	0.514
	CAD measurements	44.350	−1.625	26.837	8.088	0.514

*SEM, standard error of the mean; CAD, computer-aided detection system.*

In addition, Pearson correlation coefficients comparing the measurements of the imaging physician and the computer are shown in Table 2. The correlation coefficients for Tönnis angle, Sharp angle, and CE angle were 0.902, 0.887, and 0.902, respectively, and were statistically significant.

**TABLE 2:** Pearson correlation coefficients comparing physician with computer-aided measurement.

	*r*	*P*
Tönnis	0.902	0.000
Sharp	0.887	0.000
CE	0.902	0.000

### Analysis of Tönnis Angle Results

**FIGURE 7:**
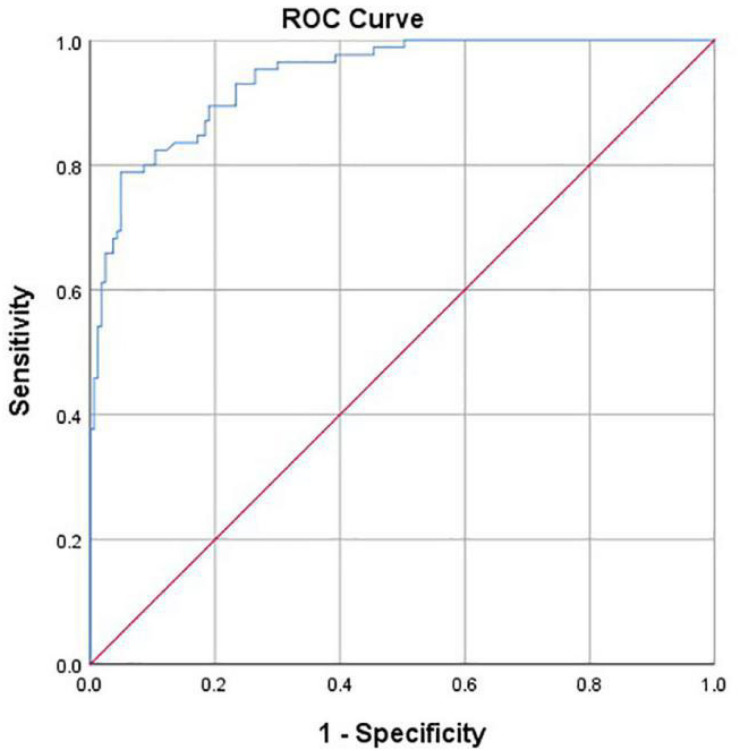
Receiver operating characteristic (ROC) curve obtained by the computer-aided system based on Tönnis angle for the development of the hip joint. AUC = 0.940.

Hip joint developmental assessments based on Tönnis angle measurements are shown in Table 3. In all 163 cases with normal development of the hip joint, computer-aided detection identified 155 cases with normal development, accounting for 95.1%. The other eight cases were misdiagnosed as dysplasia, accounting for 4.9%. In addition, of all 85 cases of dysplasia of the hip joint, computer-aided detection correctly identified 66 cases (77.7%). Another 19 cases were misdiagnosed as normal development, accounting for 22.3%. If dysplasia is taken as the positive criterion, the accuracy rate of the computer-aided system in assessing the development of the hip joint was 89.1%, the sensitivity rate was 77.7%, the specificity rate was 95.1%, and the false-positive rate was 4.9%. The McNemar test showed no statistical difference between the computer-aided system and the imaging physician in assessing the development of the hip joint (*P* = 0.052). The AUROC curve value was 0.940 (Figure 7). When the ROC threshold was chosen to be 9.5, the value of the computer-aided system to determine the development of the hip joint was highest.

**TABLE 3:** Computer-aided detection system assessment results using Tönnis angle.

		CAD judgment		Summation
		Normal	Dysplasia	
Reference standard	Normal	155	8	163
	Dysplasia	19	66	85
Summation		174	74	248

*CAD, computer-aided detection system.*

### Analysis of Sharp Angle Results

**FIGURE 8:**
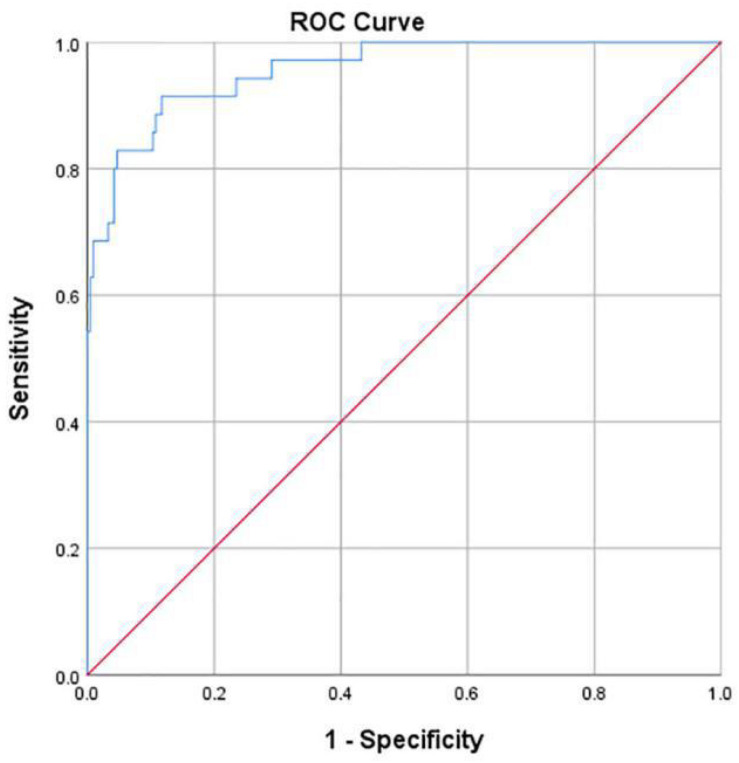
ROC curve obtained by the computer-aided system based on Sharp angle for the development of the hip joint. AUC = 0.956.

Hip joint developmental assessments based on Sharp angle are shown in Table 4. Among 213 patients with normal hip joint development, 204 patients (95.8%) were correctly identified by computer-aided detection. The other nine cases were misdiagnosed as dysplasia, accounting for 4.2%. Among the 35 cases of dysplasia of the hip joint, 27 cases (77.1%) were correctly identified by computer-aided detection. The other eight cases were misdiagnosed as normal development, accounting for 22.9%. With dysplasia as the positive criterion, the accuracy rate, sensitivity, specificity, and false-positive rate of the computer-aided system were 93.1, 77.1, 95.8, and 4.2%, respectively. The McNemar test showed no statistical difference between the computer-aided system and the imaging physician in assessing hip joint development (*P* > 0.05). In addition, the κ value was 0.721 (*P* < 0.001), which indicates that the computer-aided system was consistent with the imaging physicians’ assessment on the development of the hip joint. The AUROC curve value was 0.956 (Figure 8). When the ROC threshold was selected to be 43.545, the value of the computer-aided system to determine the development of the hip joint was highest.

**TABLE 4:** Computer-aided detection system assessment result using Sharp angle.

		CAD judgment		Summation
		Normal	Dysplasia	
Reference standard	Normal	204	9	213
	Dysplasia	8	27	35
Summation		212	36	248

*CAD, computer-aided detection system.*

### Analysis of CE Angle Results

Hip joint developmental assessments based on CE angle are shown in Table 5. Among 174 patients with normal development of hip joint, 154 (88.5%) were correctly identified by computer-aided detection. Among the 27 cases of dysplasia of hip joint, 23 cases (85.2%) were correctly identified by computer-aided detection. In 47 cases of marginal dysplasia of the hip joint, computer-aided detection correctly identified 27 cases, accounting for 57.4%. The accuracy of the computer-aided system in assessing the hip joint development based on the CE angle was 82.3%.

**TABLE 5:** Computer-aided detection system assessment results using center-edge (CE) angle.

		CAD judgment			Summation
		Normal	Dysplasia	Borderline	
Reference	Normal	154	2	18	174
standard	Dysplasia	0	23	4	27
	Borderline	6	14	27	47
Summation		160	39	49	248

*CAD, computer-aided detection system.*

In addition, after the measurement of the hip joint’s CE angle, assessments were made based on whether the joints were normal or showed dysplasia/borderline dysplasia, as shown in Table 6. Among 174 patients with normal development of hip joint, 154 (88.5%) were correctly identified by computer-aided detection. Of the 74 cases of dysplasia of the hip joint, 68 (91.8%) were correctly identified by computer-aided detection. The McNemar test showed statistical differences between the computer-aided system and the imaging physician in assessing the development of the hip joint according to the classification with three classes (*P* = 0.004) or two classes (*P* = 0.009).

**TABLE 6:** Computer-aided detection system assessment results using CE angle.

		CAD judgment		Summation
		Normal	Borderline and dysplasia	
Reference	Normal	154	20	174
standard	Borderline	6	68	74
	and dysplasia			
Summation		160	88	248

*CAD, computer-aided detection system.*

**FIGURE 9:**
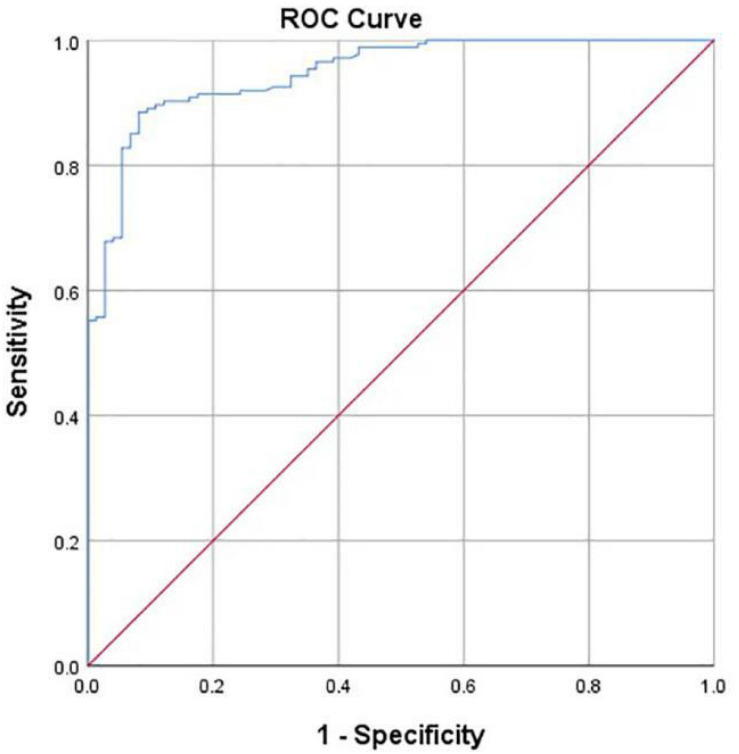
ROC curve obtained by the computer-aided system based on CE angle for the development of the hip joint. AUC = 0.948.

For the dichotomous classification, the AUROC curve value was 0.948 (Figure 9). When the ROC threshold was selected to be 24.905, the value of the computer-aided system to determine the development of the hip joint was highest.

## Discussion

The hip joint is one of the largest active joints in the human body, and its ability to sustain normal daily activities determines people’s quality of life. Hip dysplasia has long been a risk factor for joint degeneration and pain (Wilkin et al., 2017). In recent years, the field of adolescent hip joint surgery has developed rapidly, for the main purpose of preventing degenerative osteoarthritis caused by dysplasia. The American Academy of Pediatrics has recommended regular physical examination in infancy (Yang et al., 2019), in the hopes of giving patients appropriate clinical management in the early stage of dysplasia. At present, imaging examination methods for assessing the development status of the hip joint are becoming increasingly mature, including x-ray, CT, and MRI, among which x-ray is often the first choice for clinicians due to its advantages of low cost, short time, and high convenience. Therefore, if both clinicians and imaging physicians can fully understand how to perform x-ray diagnosis of hip dysplasia, unnecessary additional imaging can be reduced, which not only can relieve the working pressure of medical units, but also can reduce the economic burden of patients.

### Parameters of Hip Joint

Many parameters can be used to assess stable hip joint development, such as the CE angle, Tönnis angle, Sharp angle, acetabular depth, integrity assessment of the Shenton line, and pelvic height measurement. Li et al. (2016) demonstrated that with hip dysplasia on one side, ipsilateral pelvic height would continue to decline, which results in the continuous increase of the bilateral height difference of the pelvis and pelvic asymmetry and that the degree of hip dysplasia was positively correlated with the degree of pelvic asymmetry.

Acetabular dysplasia is a major cause of osteoarthritis; nearly half of all patients with acetabular dysplasia eventually develop secondary osteoarthritis (Cooperman et al., 1983). Rhee et al. (2011) showed that the Shenton line could be used as a reliable imaging indicator to determine the presence or absence of femoral head dislocation. An incomplete Shenton line not only indicated the presence of femoral head dislocation, but also highly suggested acetabular dysplasia. In addition, their study (Rhee et al., 2011) indicated that the reliability of the Shenton line was very good – the κ value could be 0.9 or greater, surpassing most other radiological parameters. Although studies of this kind have shown that the integrity of the Shenton line can be used as a good assessment indicator of hip dysplasia with high reliability, in some patients in the early stage of hip dysplasia, the Shenton line is still intact without dislocation; therefore, other parameters, such as angle measurement, must be used to comprehensively assess the hip joint development.

Center-edge angle and Tönnis angle are commonly used as measurement indices of the hip joint. The typical definitions of dysplasia are CE angle <20° and Tönnis angle >10°, which, respectively, represent over-shallow acetabular development and over-upward inclination (Kosuge et al., 2013), which results in uneven forces on the hip joint. Henak et al. (2014) established a finite element model of the hip joint with different developmental conditions and analyzed the elastic mechanics of model hip joints with normal development and those with dysplasia. The results showed that the pressure of the dysplastic hip joint was 2.8 to 4.0 times that of the normal hip joint.

In this study, hip dysplasia was defined as CE angle <20°, Tönnis angle >10°, and Sharp angle >45°. However, when the CE angle is between 20 and 25°, it is considered as borderline dysplasia. Mabuchi et al. (2006) defined dysplasia as CE angle <20°, Tönnis angle >15°, and Sharp angle >45°. Feldman et al. (2010) assessed dysplasia as CE angle <20° and Tönnis angle >10°. Although there is some controversy about the critical value of hip dysplasia based on Tönnis angle, Pereira et al. (2014) found that 62% of patients with CE angle <20° had a Tönnis angle >15°, and 72% of patients with borderline dysplasia (20° < CE < 25°) had Tönnis angles >10°. This suggests that when the Tönnis angle is between 10 and 15°, a considerable number of patients have dysplasia. Therefore, in this study, when defining dysplasia using Tönnis angle, 10° instead of 15° is used as the critical value.

The tilt of the pelvis or the definition of the acetabular margin (whether or not to include osteophytes) may have an impact on angle parameters measured at the hip joint. Lee et al. (2011) showed that pelvic tilt had little influence on the measurement results of CE angle and Sharp angle; however, with different acetabular margin definitions, the CE angle and Sharp angle were more variable. In particular, CE angle measurements significantly increased when osteophytes were included in the definition of the acetabular margin, which may lead to underestimation of acetabular dysplasia in epidemiological studies. However, patients in this study showed no significant osteosis or osteophyte formation at the acetabular margin, so this factor would not have a substantial impact on the results.

### Applications of Computer-Aided Detection Systems

Artificial intelligence in radiology uses data to provide computers with the ability to learn (Wang and Summers, 2012). Automatic analysis of medical images through machine learning is a rapidly growing field, and an important subset of machine learning is artificial neural networks, which is by far the most used and successful technique in radiology. The most common use of artificial neural network systems with images is for computer-aided detection (Choy et al., 2018; Fazal et al., 2018). Computer-aided diagnosis consists of two parts: computer-aided diagnosis and computer-aided detection, which have been used in image analysis for a variety of clinical diseases. For example, in x-rays of breast lesions, computer-aided diagnosis has been proven to be effective (Dromain et al., 2013). Katzen and Dodelzon (2018) reported that the sensitivity of diagnosis using mammogram images increased from 80.4 to 84% after the addition of computer-aided diagnosis. In addition, compared with those by a doctor, the integration of computer-aided diagnosis improved the detection rate of pulmonary nodules with x-ray or CT (Das et al., 2006; Yuan et al., 2006; Kligerman et al., 2013).

In fact, deep learning has been applied in image segmentation; the neural network model represented by Unet shows good performance in medical image segmentation. Wei et al. (2020) used the improved network model based on Unet to segment the femur to calculated caput-collum-diaphyseal angle and achieved relatively good results. Li et al. (2019) automatically identified the outer margin of the acetabulum and the lower margin of teardrops through the Mask-RCNN network model for the automatic measurement of Sharp angle. Both methods use neural networks in measuring hip joint angles and obtain good model performance. However, Wei et al. (2020) only segmented the femur and did not automatically segment the acetabulum simultaneously. In addition, Li et al. (2019) measured Sharp angle by identifying key points such as the outer edge of the acetabulum and the lower edge of teardrops; however, if the number of key points is too small, the model may be over-fitted as occurred in their initial research. Li et al. later improved the performance of the model by increasing the total number of key points to calculate the coordinate values of the required target points. More importantly, the above studies were each limited to the measurement of a single angle type. For assessments of hip joint development, only one angle is not sufficient. Therefore, we proposed a method to semiautomatically extract Tönnis, Sharp, and CE angles simultaneously to provide clinicians with more information.

The accuracies of computer-aided detection in making developmental assessments of the hip joint based on Tönnis angle, Sharp angle, and CE angle were greater than 80%. These research results also confirm the views expressed by Fazal et al. (2018). The arrival of computer-aided programs indicates that artificial intelligence will be widely integrated into radiology.

### Characteristics and Shortcomings of This Study

There are various clinical indicators to assess hip joint development. In this study, the imaging physician and the computer-aided system were used as a comparison, and three common indicators of the hip joint (Tönnis angle, Sharp angle, and CE angle) were measured. Computer-aided measurement can automatically measure different angles at the same time from the outline of the hip joint; however, traditional manual measurement can measure only a single angle at a time, and when measuring different angles, some bone markers or linear structures may need to be reused, which is more time consuming. The measurements of Tönnis angle, Sharp angle, and CE angle by the imaging physician and the computer were highly correlated, with correlation coefficients of 0.902, 0.887, and 0.902, respectively. The accuracy and sensitivity of the computer-aided detection system for the semiautomatic measurement of Tönnis angle were 89.1 and 77.7%, respectively. The accuracy of semiautomatic measurement of Sharp angle was 93.1%, and the sensitivity was 77.1%. The accuracy of the CE angle measurement was 82.3%, although the accuracy was only 57.4% for the sole measurement of the hip joint with borderline dysplasia (20° < CE < 25°); however, considering that the definition of borderline dysplasia was too narrow, this does not deny the achievement of this study.

There are some limitations. First, the measurement of reference standard was completed by one imaging physician. To compensate for this deficiency, a chief physician with more than 20 years’ working experience provided guidance and training during the parameter measurement, and reliability analysis was conducted on the measurement results, which showed that the measurement results of the imaging physician were highly reliable. Second, 248 hip joints were included in this study, which is a small sample size. Additionally, the results showed a slightly higher degree of dispersion. With the expansion of sample size, this deficiency can be improved. Third, our current method is semiautomatic, and the main drawback of the method is the need to manually trace the bone profile of the hip joint. It is currently difficult to realize the automatic segmentation of acetabulum. Therefore, complicated acetabular imaging, coupled with a small sample size, leads to the failure of the existing neural network. In this study, contouring is the basis for measuring all angles, so in the future, automatic segmentation of hip joint contour is needed to realize fully automatic measurement of different angles of hip joint. Fourth, previous studies (Pereira et al., 2014) divided patients with hip dysplasia into three groups according to the CE angle: 21° < CE < 25° was mild dysplasia, 11° < CE < 20° was moderate dysplasia, and CE < 10° was severe dysplasia. However, this study did not further classify the severity of dysplasia in patients with CE < 20°. In the future, we should continue to expand the sample size, qualitatively determine the development of the hip joint into more accurate subgroups, and further evaluate the diagnostic value of computer-aided system in the assessment of hip joint development.

## Conclusion

In this study, different angle indices of the hip joint were semiautomatically extracted and measured according to the bone contours of the hip joint, and the diagnostic value of the computer-aided system when applied to different indices was analyzed. This study confirmed that the computer-aided system is highly correlated with the measurements of Tönnis angle, Sharp angle, and CE angle by an imaging physician and that the assessments had high accuracy and sensitivity. In the future, computer-aided systems are expected to contribute significantly to the developmental screening or diagnosis of the hip joint.

## Data Availability Statement

The raw data supporting the conclusions of this article will be made available by the authors, without undue reservation.

## Ethics Statement

The studies involving human participants were reviewed and approved by Ethics Committee of Zhongshan Hospital affiliated to Dalian University. Written informed consent for participation was not required for this study in accordance with the national legislation and the institutional requirements. Written informed consent was not obtained from the individual(s) for the publication of any potentially identifiable images or data included in this article.

## Author Contributions

XC, ZQ, and XZ planned the study together. YJ, GY, and BC completed the experiments. YL collected the cases. YJ and QS completed the statistical analysis. YJ wrote the manuscript. All authors contributed to the article and approved the submitted version.

## Conflict of Interest

ZQ was employed by the company Heilongjiang Tuomeng Technology Co., Ltd. The remaining authors declare that the research was conducted in the absence of any commercial or financial relationships that could be construed as a potential conflict of interest.
